# Droplets, Evaporation and a Superhydrophobic Surface: Simple Tools for Guiding Colloidal Particles into Complex Materials

**DOI:** 10.3390/gels3020015

**Published:** 2017-05-04

**Authors:** Marcel Sperling, Michael Gradzielski

**Affiliations:** Stranski Laboratorium für Physikalische & Theoretische Chemie, Institut für Chemie, Technische Universität Berlin, Straße des 17. Juni 124, Sekr. TC7, D-10623 Berlin, Germany

**Keywords:** superhydrophobic surfaces, droplets, nanoparticles, evaporation, self-assembly, self-propelling, anisometric, colloids, supraparticles, functional materials, catalysis

## Abstract

The formation of complexly structured and shaped supraparticles can be achieved by evaporation-induced self-assembly (EISA) starting from colloidal dispersions deposited on a solid surface; often a superhydrophobic one. This versatile and interesting approach allows for generating rather complex particles with corresponding functionality in a simple and scalable fashion. The versatility is based on the aspect that basically one can employ an endless number of combinations of components in the colloidal starting solution. In addition, the structure and properties of the prepared supraparticles may be modified by appropriately controlling the evaporation process, e.g., by external parameters. In this review, we focus on controlling the shape and internal structure of such supraparticles, as well as imparted functionalities, which for instance could be catalytic, optical or electronic properties. The catalytic properties can also result in self-propelling (supra-)particles. Quite a number of experimental investigations have been performed in this field, which are compared in this review and systematically explained.

## 1. Introduction

**Figure 1:**
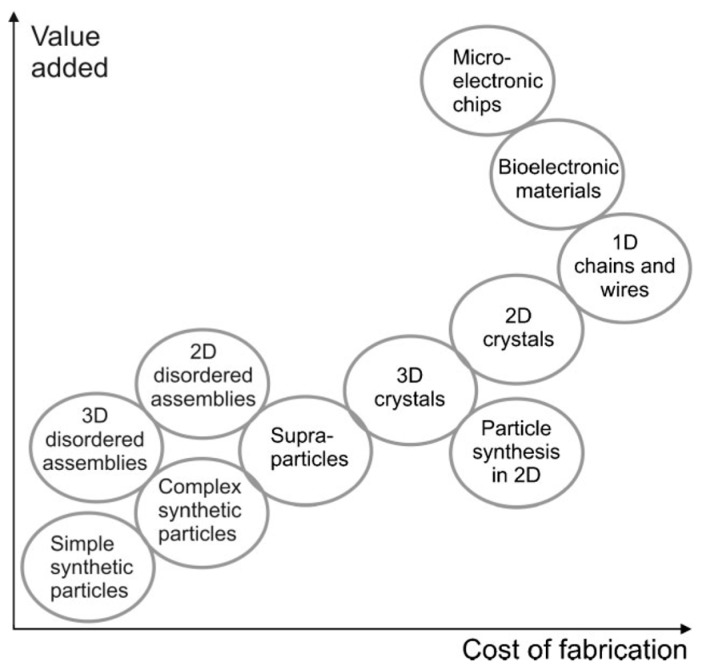
Hypothetical qualitative estimation of the value-to-price ratio for different products gathered by colloidal assembly. Adapted with permission from [4] (p. 7), Copyright 2009 Wiley.

Self-assembly of colloids into bigger particles (from µm to mm) has been in the focus of colloid and material research for some time, as it allows for the fabrication of materials providing various functionalities [1,2,3]. Their production can be accomplished by the utilization of several different techniques that have been developed over the last few years, such as sedimentation, evaporation, adsorption, external force fields, bio-recognition or surface and droplet templating, which have been summarized elsewhere [4,5,6]. Especially, considering the ratio of estimated fabrication costs vs. the added value in terms of provided functionality is a crucial aspect, when it comes to potential applications or scale-up for industrial production. Hypothetically, this ratio has been estimated by Velev and Gupta as shown in Figure 1 [4]. They were also relating principally available techniques, also those mentioned above, to the accessible dimensionality (1D, 2D or 3D) and scalability of the procedure. One may state that usually, the lower cost processes are favored for scaling up [4]. Besides the applied technique, also the quality of materials plays an important role. When considering dispersed particles, especially on the micron- or even nano-meter scale, the efforts needed for their synthesis may increase dramatically when seeking a high degree of monodispersity, as for instance required in photonic applications [7]. In order to produce high quality photonic crystals [8], a defined control of inter-particle spacing is needed to achieve a high sensitivity for the desired diffracted wavelength. Thus, this sensitivity depends on the fluctuations in particle size as that determines the quality of the corresponding crystal lattice. This correlation of in-depth lattice spacing with the resulting diffracted wavelength is expressed by Bragg’s law [9]. Moreover, besides pure size aspects of the assembling particles, also their shapes, either static [10,11,12,13] or dynamic [14,15] can be of major importance.

There exist multiple ways of producing colloidal assemblies, such as supraparticles, that differ with respect to dimensionality, cost and scale. Many of these ways have been reviewed by Velev and Gupta [4]. In general, all of these methods start from confining the colloidal building blocks within a droplet (or generally a container). This could for example be done using emulsion droplets, where the solubilized liquid becomes removed by heating and other approaches. However, our main focus in this work will be set on evaporation-induced self-assembly (EISA) on super-repellant solid surfaces, typically superhydrophobic ones. In the following sections, we shall give a detailed overview of the basics of this technique, in terms of the materials applied and the condition parameters to be set, as well as the most recent materials fabricated by it.

In that context, EISA is a particularly simple and therefore appealing approach, in which one simply evaporates the solvent of a colloidal dispersion leading to a situation where the ingredients become more or less arranged during the drying process [13,14], thereby forming self-assembled nanostructures in a simple way. In this fashion, one may access structured thin films [16,17], but also supraparticles by the preparation from droplets deposited on a solvophobic surface. Probably the most common version of the latter are superhydrophobic surfaces applicable to water droplets [18,19,20,21,22].

In the scope of this review, we will present recent achievements in the field of supraparticle fabrication using aqueous suspension droplets dispensed and dried on superhydrophobic surfaces, i.e., using EISA and droplet templating. After some fundamental discussion of EISA and the droplet templating method, as well as superhydrophobic surfaces, we will show possible ways to create supraparticles of various shapes, hierarchical structures (like Janus-type) and functionalities. Furthermore, we will give some general discussion about applications for supraparticles that have recently been developed. It may be noted that in our review we focus on large supraparticles in the size range of hundreds of µm or even mm, neglecting the abundant work on smaller-sized assemblies.

## 2. Evaporation-Induced Self-Assembly and the Droplet Templating Method

The concept of evaporation-induced self-assembly (EISA) uses the controlled removal of a volatile component to trigger the defined aggregation of contained materials. This aggregation can either occur due to continuously limiting the available space for the dispersed components or simply due to their increasing concentration. One example for the latter can be found within mixed surfactant systems, in which the starting solution has a concentration below the cmc (critical micelle concentration), which is surpassed during subsequent evaporation of the solvent. This leads to spontaneous formation micellar aggregates [23,24]. In this article, we shall focus on systems of colloidal dispersions containing insoluble particles, which are forced to assemble thereby potentially promoting colloidal crystallization via EISA [5,8,25].

**Figure 2:**
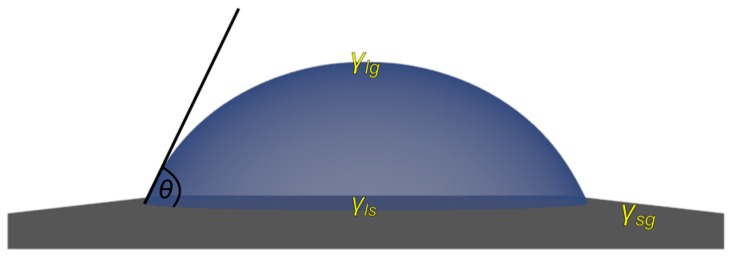
Scheme describing the contact angle (CA) *θ* of the liquid droplet on a solid substrate and its relation to the different interfacial tensions between the solid (*s*), liquid (*l*) and gas (*g*) phase, as related to each other by Equation (1).

At first glance, a simple system to do so is represented by droplets with suspended colloidal components. These droplets can either float in an immiscible second liquid, e.g., water droplets in fluorinated oil, or be dispensed on a solid surface. Considering the latter, the resulting assemblies strongly depend on the type of surface used, as the interaction between the droplets’ liquid phase and the solid substrate may vary substantially. Therefore, the wetting properties constitute the predominant influencing parameter, characterized by adhesion forces and contact angle (CA). The CA for a droplet deposited on a solid substrate depends on the interfacial tensions between the different phases (Figure 2) and is given by Young’s equation (Equation (1)), with θ being the CA and γ the interfacial tension between the solid (s), gas (g) and liquid (l) phases, respectively.

(1) $$\cos\theta =\frac{\gamma_{sg}-\gamma_{sl}}{\gamma_{lg}}$$

**Figure 3:**
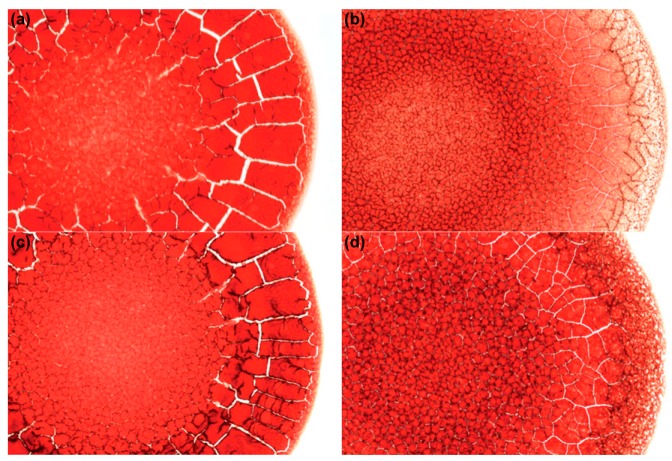
Blood samples dried on a glass surface from: (**a**) 27-year-old healthy woman; (**b**) a person with anemia; (**c**) a 31-year-old healthy man; (**d**) a person with hyperlipidemia. Adapted with permission from [30] (p. 90). Copyright 2011 Cambridge University Press.

A well-known phenomenon related to drying suspension droplets on solid surfaces at low CA is the so-called “coffee-ring effect” [26,27]. Closer investigation of the leading mechanism in these drying spherical cap droplets revealed that convective liquid transport occurs from the center apex down and outwards to the three-phase contact line (TPCL). This preserves the geometry upon strong evaporation at the droplet edge, which is driven by pinning of the TPCL due to the contained particles adhering to the surface, thus prohibiting transversal shrinkage during evaporation. Consequently, colloidal material accumulates and finally precipitates at the TPCL, leaving a solid ring-like pattern. Such a pattern can be quite characteristic for the dried sample, e.g., depending on its ionic strength [28,29]. This effect can for example be of interest for diagnostic purposes when investigating dried droplets of blood, as shown in Figure 3 [30,31]. 

**Figure 4:**
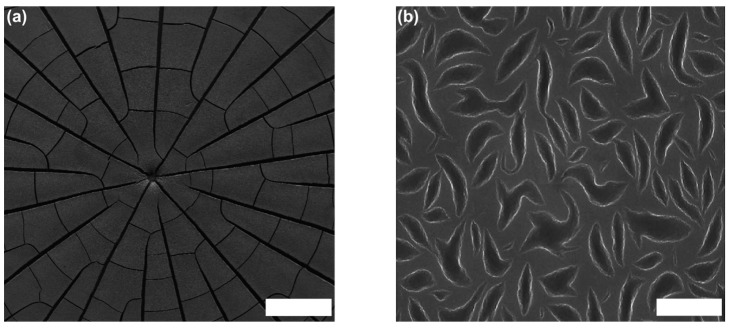
(**a**) SEM image of dried drops for a ratio of silica NPs (diameter: 10 nm)/DNA (20,000 bp) ratio 1:0.5. The scale bar is 200 µm; (**b**) HR-SEM images of dried drops for an NP/dsDNA ratio of 1:0.5. The scale bar is 2 µm. Adapted with permission from [32] (p. 3663). Copyright 2014 Springer Nature.

The formation of “cracks” such as seen in Figure 3 is always an indication of rather small CAs and pronounced pinning of the droplets on the substrate. In that context, it might be mentioned that such cracks have also been observed very pronouncedly for drying droplets containing mixtures of DNA and small silica nanoparticles (Figure 4). The final solid films showed interesting surface patterns with unique shapes, which are induced by local segregation of the two colloidal components of DNA and silica [32]. For more complexly-composed colloidal mixtures, such local segregation is a further complication that may arise in the EISA process.

**Figure 5:**
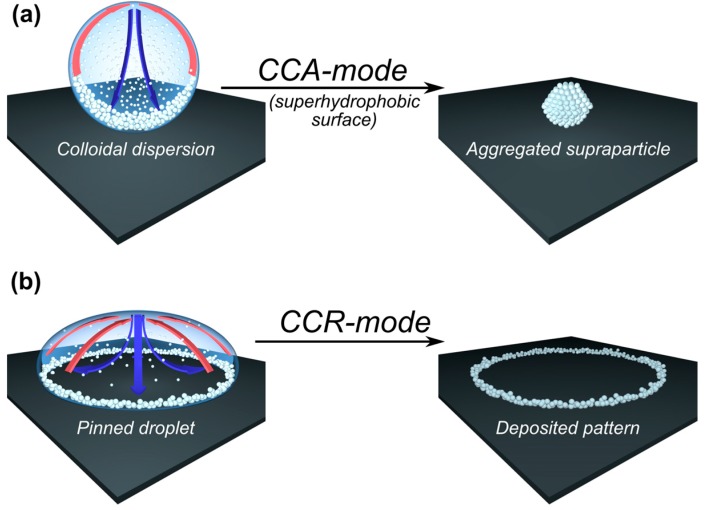
Schematic description of the different modes observed for a drying droplet on a solid substrate, with the mass flow of cooled water (blue arrows) and that due to the interfacial tension gradients (Marangoni flow; red arrows) being indicated for: (**a**) constant contact angle (CCA) mode, as observed on most superhydrophobic surfaces; (**b**) constant contact radius (CCR) mode.

The process of coffee-ring formation is counterbalanced by the Marangoni effect [33,34,35]. This effect describes convection processes arising from thermally-induced gradients in the droplet’s interfacial tension (also called Bénard–Marangoni convection). In addition, convectional flow simply arising from a decrease in surface temperature due to evaporation is observed, which then in turn causes density differences (see also Figure 5). Usually, the occurrence of coffee-ring deposits indicates stronger evaporation closer to the TPCL and low or even absent Marangoni stresses [36]. It is also reported that experimentally, Marangoni convection in water droplets is often lower than expected from theoretical simulations, which can be related to the surface contaminants enriching at the water interface with ease and, hence, lowering the interfacial tension [35,37]. Generally, thermal conductivity and the latent heat of evaporation are important parameters that determine the extent of Marangoni flow in an opposing fashion due to the generation of a thermal gradient, but also concentration gradients of ingredients, e.g., surfactants, may induce such convective flow. However, for the case of EISA, the thermal gradient-induced Marangoni effect should play the predominant role. Furthermore, the Marangoni convective flow may also completely reverse the coffee-ring effect, as reported for octane droplets on glass [35].

The basic concepts of microfluidic flow inside sessile droplets are usually discussed for the case of a pinned droplet contact line, i.e., the “constant contact radius” (CCR) mode. Of course, one may also observe the complementary case, in which the droplet’s contact line successively recedes at constant CA due to the absence of any pinning effects, i.e., “constant contact angle” (CCA) mode. The details of the evaporation process for the two different modes (CCR and CCA) are described in Figure 5. In contrast to the coffee-ring effect, Marangoni convection will occur for both CCR and CCA modes [33,34]. It might be added here that it is also possible to use electric fields to manipulate the wetting behavior of a sessile droplet [38,39,40].

The provided mode of wetting directly controls the kinetics of the evaporation process, which has been semi-empirically analyzed by Picknett and Bexon [41]. It was already postulated by Maxwell that the rate of mass loss $\frac{dm}{dt}$ of an evaporating sessile droplet is attributed to the product of the vapor diffusion coefficient D in air and the difference of the vapor concentration at the droplet surface $c_{s}$ (which can be approximated by the saturation vapor concentration), its value in the surrounding $c_{\infty }$, as well as the shape-dependent electrostatic capacitance C (Equation (2)) [42].

(2) $$\frac{dm}{dt\;}=4\pi DC\left(c_{s}-c_{\infty }\right)$$

Starting Snow’s series [43], Picknett and Bexon used a polynomial evaluation for equiconvex lenses, being similar to those of sessile droplets at different CAs. As a result, an empiric expression was obtained giving C/r as a function of the CA, represented by f(θ), with r being the droplet radius [41]. In this way, one accounts for the interfacial blockage by the solid surface, which prohibits free vapor diffusion and thus reduces local evaporation. For 0.175≤θ≤π, this translates into Equation (3).

(3) $$f\left(\theta \right)=C/r\;=0.00008957+0.6444\;\theta +0.1160\;\theta^{2}-0.08878\;\theta^{3}+0.01033\;\theta^{4}$$

At this point, it also becomes clear that the mode of evaporation (CCR or CCA) clearly affects the evaporation as for the case of CCA f(θ) remains constant, while for CCR, the changing CA will also have an effect. Now, for the case of a constantly-receding contact-line, i.e., in the absence of droplet pinning, Equation (2) translates to a simple expression (Equation (4)) for the V(t)-function, with $V_{0}$ being the initial volume and $t_{\mathrm{total}}$ the total drying time. However, it might be noted that in recent investigations on sessile droplets of aqueous saline solutions (CAs between 2° and 50°), high salt concentrations and small contact angles showed significantly lower evaporation rates than expected from simple diffusion-controlled evaporation. Particle tracking experiments proved that this lower evaporation has to be attributed to the Marangoni effect [44]. In contrast, it has been observed that the presence of nanoparticles increases the evaporation rate of aqueous droplets, as observed for anthraquinone nanoparticles (diameter: 285 nm) on a hydrophobized silica wafer (CA: 80°) [45]. Furthermore, for evaporating droplets with an initial CA larger than 90°, one may start with a CCA mode, then switch to a CCR mode and end the evaporation in a mixed mode [46]. In general, it may be concluded that the description of the evaporation processes of droplets can become quite complicated, especially for more complex geometries. Moreover, the change in composition of the drying droplet may also have a substantial impact on the interfacial properties and, hence, on the evaporation process.

(4) $$V\left(t\right)=V_{0}{\left(1-\frac{t}{t_{\mathrm{total}}}\right)}^{\frac{3}{2}}$$

Obviously, in order to produce defined assemblies of micro- or nano-particles, which after production can be easily isolated from the surface, one will preferentially use surfaces with low adhesion forces, thus avoiding pinning of the liquid. Superhydrophobic surfaces represent a nice, biomimetic example for this purpose. Due to their large CA and, mostly, low adhesion forces, they represent an ideal substrate for the preparation of particle assemblies that allow for easy separation after drying. Hence, for the sake of completeness the basic concept of superhydrophobic surfaces will be discussed in the following section.

## 3. Superhydrophobic Surfaces

Superhydrophobic surfaces represent a class of biomimetic, nanostructured materials, where usually the term “superhydrophobicity” accounts for water contact angles (WCA) larger than 150° [18,19,20,21,22]. A common example from nature for water super-repellency is the “lotus-effect” [47] as seen for the leaf of sacred lotus [48], which also has been mimicked by artificial fabrication [49]. In general, it can be concluded that it is the combination of chemical hydrophobicity paired with surface texture on the micro- or even nano-scale, which is essential for achieving such high WCAs. Consequently, the main challenge for the artificial preparation of superhydrophobic surfaces is the introduction of surface roughness on the nm to µm scale in hydrophobic surfaces. A large variety of different technical approaches has been developed over the past years to address this challenge [50], and the bare number of publications available in the field of superhydrophobic surfaces is a statement of the success of this concept, as well as its relevance for applications. This is one reason why scientists’ curiosity even pushed the development further towards the creation of superamphiphobic surfaces, capable of efficiently repelling both oil and water [51,52]. Starting Young’s equation (Equation (1)), this is quite remarkable when considering the large range of interfacial tension values covered when comparing water to typical oils. 

### 3.1. Wetting Modes

**Figure 6:**
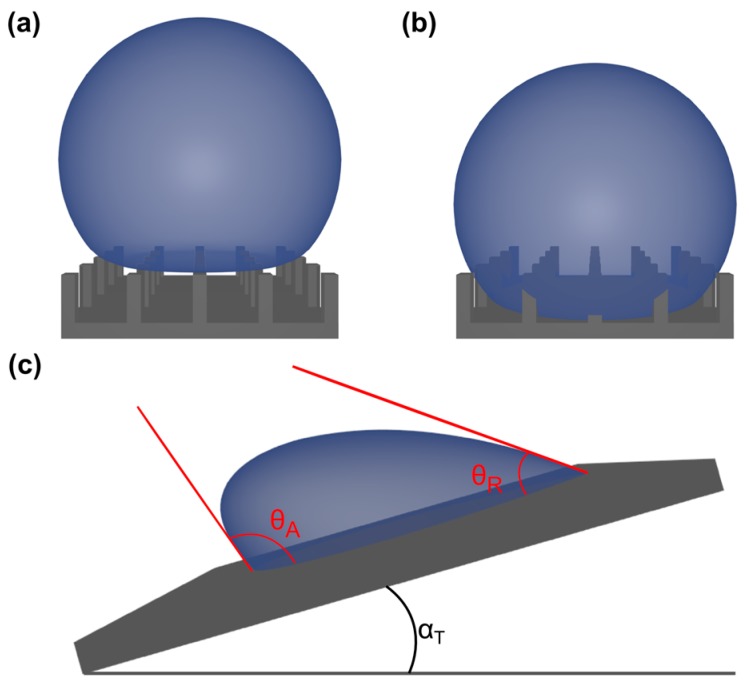
Wetting of superhydrophobic surfaces: (**a**) Cassie state with entrapped air within the surface grooves; (**b**) Wenzel state with liquid filling the grooves. Dynamic wetting strongly depends on the prevailing mode of (**a**) versus (**b**): (**c**) the difference of advancing $\left(\theta_{A}\right)$ and receding $(\theta_{R}$) CA is the measure of the surface hysteresis and droplet adhesion.

For superhydrophobic surfaces, a rough surface topography is essential to amplify hydrophobicity in order to reach WCAs of greater than 150°. Thus, the solid surface will consist of grooves on the micro- to nano-scale, where their ratio is proportional to surface roughness. Consequently, when depositing a water droplet on such a surface, one may imagine two limiting cases: either the liquid is sitting on top of the grooves, i.e., air stays entrapped within the grooves or the liquid is penetrating the same. The former state is known as Cassie’s (Cassie–Baxter) [53] and the latter as Wenzel’s [54] mode of wetting, as shown in Figure 6a,b, respectively. In the Cassie–Baxter state, the droplet is situated on top of the surfaces structure, while air stays entrapped within the interspacing provided by surface roughness (Figure 6a). Hence, at these regions, instead of wetting the surface, the droplet is in contact with air, which is the most hydrophobic (solvophobic) medium. Again, considering Young’s equation (Equation (1)), this then favors a higher CA due to $\gamma_{sl}=\gamma_{lv}$. Note that for most polymeric or waxy substrates $\gamma_{sl}$ is about equal to 30–50 mN/m, which is substantially lower than $\gamma_{lv}$ (72.8 mN/m at 25 °C). Therefore, maximizing the fraction of interspatial air pockets entrapped within the solid surface leads to CAs approaching 180°. In contrast to that, in the Wenzel state, the droplet penetrates the interspatial volume and fully wets the surface (Figure 6b).

Another important quantity is the CA hysteresis, especially when considering the self-cleaning effect of superhydrophobic surfaces. CA hysteresis is determined by the difference of the advancing ($\theta_{A}$) and receding ($\theta_{R}$) CA, as shown in Figure 6c. Low hysteresis equals a low tilting angle ($\alpha_{T}$) being necessary to cause the droplet to roll off the surface. Accordingly, paired with low adhesion forces, these rolling droplets can collect dirt particles from the surface, thereby promoting self-cleaning, as known from many plant leaves. This effect has been the inspiration for numerous materials and applications [55], such as self-cleaning and dirt- and water-repellent coatings used on tiles, roofs or panels (for instance, in bathrooms or kitchens). Using such coatings on glass leads to the “anti-fog” effect, while also antimicrobial coatings based on superhydrophobic surfaces have been investigated [55]. 

Usually, CA hysteresis stays lower for droplets in the Cassie–Baxter as compared to the Wenzel state. However, this does not automatically imply low roll-off angles for the Cassie–Baxter state. Moreover, the roll-off angle depends on the pinning forces at the contact area between solid and liquid [56]. Generally, it may be stated that for supraparticle formation by EISA, the Cassie–Baxter state with a CA as high as possible is to be preferred. 

### 3.2. Production of Superhydrophobic Surfaces

For the preparation of superhydrophobic surfaces, there exists a large range of different approaches, which also have been reviewed comprehensively in recent times [57,58]. Accordingly, we refrain here from describing these processes in further detail, but instead will briefly describe two typical approaches that also have been successfully employed in our work. One of them employs superhydrophobic surfaces produced by an electrochemical deposition (ECD) method [59]. The other is a soot-based method comprising a chemical vapor deposition (CVD) leading to surfaces that can either be rendered superhydrophobic or even superamphiphobic, the latter meaning super-repellant for water and oil [51].

ECD methods usually employ an electrochemical potential to deposit dissolved material from solution onto a surface substrate. This usually leads to a uniform and highly porous surface coverage. If the material deposited is not hydrophobic enough by itself, further chemical modification may serve to complete superhydrophobic surface formation. For example, a suitable approach was reported by Gu et al. utilizing activated copper surfaces. Here, activation usually just means surface polishing to remove the passivation layer of copper oxide or sulfide (typical reactions, when left in air) [59]. When immersed in a silver nitrate solution, a black precipitation layer of silver is formed on the copper surface. Thus, if choosing the appropriate time of immersion and silver concentration, highly porous, coral-like structures are achieved within the solid silver layer. Further functionalization with an aliphatic thiol, such as 1-dodecanethiol, then leads to a well-performing superhydrophobic surface with a CA above 150° and low CA hysteresis [59].

Another method well-known to most of us from childhood days is that of covering a heat-stable surface, such as a spoon, with soot by holding it into the flame of an ordinary candle. Unluckily, the adhesion of the as-deposited carbon layer is very poor, which results in its immediate rupture when exposed to a rolling water droplet (similar to the self-cleaning effect of lotus leaves [48]). Hence, Deng et al. searched a way to preserve the structure of the soot, while at the same time increasing its stability [51]. In order to do so, they covered the soot surface layer with a robust silica shell using CVD of a silica precursor (like tetraethyl-orthosilicate (TEOS)). After subsequent removal of the soot using calcination at high temperatures and functionalization with an appropriate aliphatic silane via CVD of a corresponding precursor, as a result, a superhydrophobic or, in the case of a fluorinated silane, a superamphiphobic surface could be achieved. Surfaces of this kind provide a CA above 150° and low CA hysteresis. Another aspect attributing quite some elegance to this method is the fact that the application of this layer can occur on any surface able to resist the temperatures used for the calcination step. Moreover, if carefully done, this surface also provides high transparency [51], which for example even allows experiments with an inverse optical microscope setup for observing the inside of the droplet from below the surface [60]. It might be noted here that the curvature of small-scale roughness was revealed to play a key role for achieving high resistance against wetting, thus being of major importance for acquiring superamphiphobicity [61].

For the sake of not losing our intended focus of this review, we will not further discuss the many other preparation methods available. Instead, the interested reader is referred to comprehensive reviews giving a nice overview of superhydrophobic surfaces and their different preparation methods that have been accomplished [19,22,50,52].

## 4. The Concept of Supraparticle Formation

Supraparticles can be created by self-assembly of colloidal components into larger, ordered arrays [62]. Such supraparticles can possess a large number of combined functionalities as they may contain many different colloidal materials of a specific nature, like proteins [63,64], photosensitive particles, such as semi-conductors [65,66], or magnetic materials [67,68]. 

**Figure 7:**
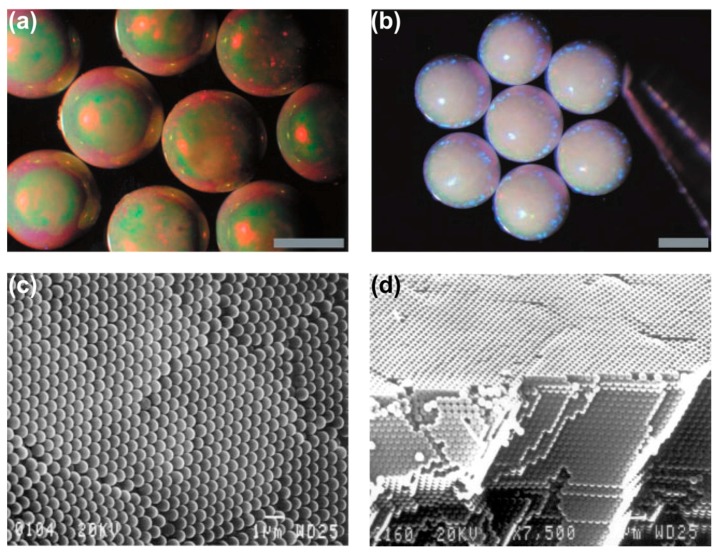
Typical examples for supraparticles prepared by drying of aqueous droplets containing polystyrene (PS) latex particles dispersed in fluorinated oil. Spherical supraparticles are formed showing different color patterns based on the size of the PS latex particles: (**a**) 270 nm and (**b**) 320 nm, scale bars = 500 µm. These patterns arise from light-diffraction due to long-range ordering of the particles as shown in (**c**) at the surface and (**d**) along the vertically-broken edge of a similar supraparticle; scale bars = 1 µm. Adapted with permission from [25] (p. 2241). Copyright 2000 Science.

With respect to their preparation, a controlled guiding of the assembly process is vital in order to create supraparticles in a defined way [69]. This means that typically, one employs droplets as the confining object within another, immiscible liquid or on a solid surface. Accordingly, besides assembling particles from freely-suspended solutions [65,70], several methods have been developed employing droplets as confining geometry. There are different methods suitable for generating droplets, such as emulsion techniques [71,72,73,74], microfluidics [75,76,77,78] or ink-jet printing [79,80,81,82], that besides precise control on the droplet size, also offer potential for scalability towards mass production. Apart from just serving as a container for the contained particles, the droplets may also actively promote the assembly process by drying induced shrinkage that constrains the colloidal components. One way to do so is dispersing aqueous colloidal suspension droplets in hydrocarbon or fluorinated oil with subsequent removal of the aqueous phase [25]. In such a setup, the position of the droplets can also be controlled using separately addressable electrodes constructed in an array allowing for defined droplet dielectrophoresis [83]. Some examples for resulting supraparticles achieved by such processes are illustrated in Figure 7 for the case of polystyrene (PS) latex particles dispersed in aqueous droplets floating in fluorinated oil.

The supraparticles shown in Figure 7a,b show a remarkably smooth and regular structure, which is also indicated by the colored appearance under top-light illumination. This color effect requires long-range ordering of the particles, which was corroborated by analyzing the colloidal structure with scanning electron microscopy (SEM). Here large arrays of regularly-ordered particles within hexagonal closely-packed planes and face-centered cubic lattices were observed, as illustrated in Figure 7c,d [25]. The driving force for this high degree of ordering was addressed by Denkov et al., who investigated 2D arrangements of monodisperse and micron-sized PS-latex particles on a solid surface [84,85]. They found that crystallization, i.e., the ordering process, always occurred when the height of the liquid’s meniscus reached the particle diameter. Furthermore it was found that this fact neither depends on the ionic strength of the suspension, nor on the surface charge or initial concentration of the particles. Thus, electrostatic and van der Waals interactions could be excluded as governing factors [85]. Instead, capillary forces between the particles caused by the menisci formed and convective particle transport within the solution were found as the predominant control parameters. This extraordinarily high degree of long-range ordering also leads to a precise control of the porosity, e.g., by tuning the size of the particles or applying additives, such as glucose [86] or DNA to drying silica suspensions [32]. Using a microfluidic setup and analyzing the time-dependent solute release profile from similar sub-mm-sized supraparticles containing 320-nm diameter PS latex microspheres using a dye, Rastogi et al. showed that such assemblies provide a permeable matrix allowing for a more homogeneous and slower dye release compared to normal pellets [87]. The internal structuring of colloidal spheres within liquid droplets also was discussed based on geometrical constraints, surface tension and the interparticle potential. Depending on the detailed conditions, one may expect the formation of colloidal clusters, colloidosomes or Pickering emulsions [88]. Here, the latter two structures are most likely to be observed upon the conditions prevalent if the second phase is another liquid, i.e., in an emulsion.

## 5. Supraparticles by EISA on Superhydrophobic Surfaces

In the following, we will focus on supraparticles obtained by drying aqueous suspension droplets on (solid) superhydrophobic surfaces. Recent work in that field has shown how simple procedures grant precise control over the shape and internal hierarchical structure in these supraparticles, thereby leading to new kinds of functional materials.

One major drawback of using a two-liquid system, as often employed, is the difficult isolation of the obtained products and their purification. This fact gave rise to the utilization of superhydrophobic surfaces. Due to their high WCA, these surfaces also provide spherical droplets and thus isometric surrounding conditions, but avoid the need of subsequent separation of a second (oil) phase from the dried supraparticles. Instead, those can readily be collected from the surface due to low adhesive forces (as evident from the high WCA and low roll-off angle). Note, that despite being superhydrophobic, these surfaces can be designed for both adhesive and non-adhesive properties by tuning pitch values and the density of micro- and nano-structures, respectively [89,90]. Using EISA on such superhydrophobic surfaces in combination with the droplet templating method, several new types of supraparticles of different structures and functionality have been produced. However, in order to be easily isolable, the droplets must not penetrate the surface texture. Consequently, the size range of supraparticles produced on such surfaces is limited to several µm [91] as a lower size limit and may reach about mm size [92,93,94,95,96,97], while for still larger droplets gravitational effects become dominant leading to their shape being no longer spherical.

In the following, we shall present recently developed methods for the creation of supraparticles of various kinds of shapes and functionalities based on their preparation on superhydrophobic surfaces. 

### 5.1. Shaping of Supraparticles

In the simple case, the shape of the droplet will directly determine that of the finally obtained supraparticle, since the contained particles remain trapped within the provided geometry, i.e., one simply templates the initial shape. Therefore, the confining geometry is retained. As this geometry on superhydrophobic surfaces is to a first order spherical (except for gravitational effects), the resulting supraparticles for the ordinary case are also of a similar shape. Hence, in order to alter the final shape of the supraparticles, one has to change the droplet geometry. For the case of aqueous droplets dispensed and floating on fluorinated oil and containing PS-latex microspheres, this was done by using additional fluorinated surfactant. This led to the formation of dimpled, red blood cell-like or even doughnut-shaped supraparticles at higher concentrations [25], which is caused by the change of the interfacial tension between the liquids promoted by the surfactant. Lastly, a pronounced deviation from spherical geometry also occurred when lowering the particle concentrations below 20%, and a continuous transition from a spherical to disc-like shape was observed [25]. 

Superhydrophobic surfaces, for the case of up to µL-volumes, also provide spherical droplets, with some slight, but mostly not substantial bottom deformation due to gravitational effects. This can be deduced from the high CA and low Eötvös or Bond number *B*_0_ (Equation (5)), which is a dimensionless number describing the relative influence of gravity and surface tension on the shape of a liquid drop. Here, Δρ represents the density difference between the droplet and its surroundings, g the gravitational constant, r the droplet radius and γ the interfacial tension [98].

(5) $$B_{0}=\frac{\Delta \rho gr^{2}}{\gamma }$$

**Figure 8:**
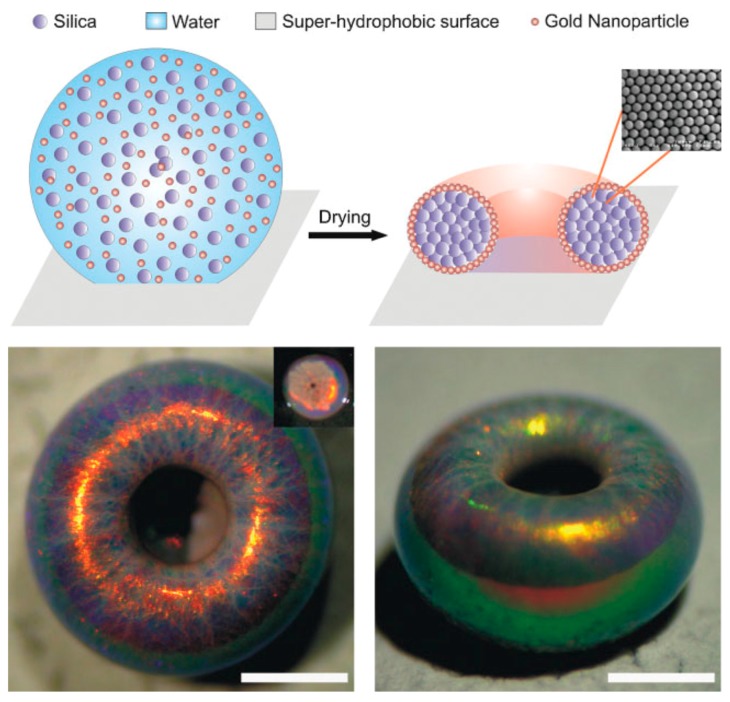
Top: Illustration of the evaporation-induced formation of “doughnut” supraparticles; the inset is showing an SEM of the particle lattice built by 330-nm diameter silica particles. Bottom: Optical micrographs of the final supraparticle from top- (left) and side-view; scale bars are 500 µm. Adapted with permission from [92] (p. 192). Copyright 2010 Wiley.

Rastogi et al. used superhydrophobic surfaces to create supraparticles of a reduced symmetry, “doughnut”-like shape, as shown in Figure 8 [92]. The morphological loss of symmetry corresponds to the geometric alteration from spherical $R_{3}$- to cylindrical $D_{\infty }$-symmetry. This interesting overall shape is accompanied by light diffraction effects that are similar as for the spherical supraparticles arising from the segregation of the different colloids contained in the supraparticle. 

It has been reported for aqueous polymer solution droplets that a thin shell of concentrated polymer is formed at the water-air interface, due to internal transport flux. This flux is directed radially outward and significantly influences the droplet’s shape upon evaporation due to increased surface elasticity promoted by the concentration gradient [29,98,99,100,101]. If this accumulation is stronger at the three-phase contact line (TPCL) than at the apex, self-pinning of the droplet occurs. This means that the transversal shrinkage is suppressed, which in turn results in exclusive shrinkage along the droplet height. Note that the origin of this self-pinning is notably different from the coffee-ring effect, where the droplets are pinned due to the surface wetting properties [26,27]. The importance of the CA for the shape of the formed dry supraparticles has been demonstrated by assembling silica microspheres of 300 nm via EISA on substrates with CAs ranging from 28°–105°. Depending on the CA, ring-like structures, doughnut-like structures or hemispheres were formed [102]. Such hemispherical assemblies could also be achieved by EISA on a substrate with a CA of 100° when employing monodisperse latex particles with diameters varying in the range of 300–1100 nm. Here, a very high degree of ordering of the latex particles was locally observed, which created pores of relatively high uniformity in theses supraparticles [103].

Large particles contained within the drying solution similarly undergo surface collection in analogy to polymers [28,60]. In the case of the doughnut silica supraparticles, the colloidal particles (330-nm diameter silica microspheres) get collected at the interface of the precursor droplets due to the shrinking surface, which is propagating faster towards the interior than can be counterbalanced by particle diffusion [92]. As the particles predominantly collect close to the TPCL, the droplets deform in a fashion that yields dimpled supraparticles for volume fractions of silica lower than 15%. However, when working at a silica volume fraction of 2.5%, the dimple evolved into a complete hole, hence yielding doughnuts. This effect vanishes when using 1000-nm instead of 330-nm silica particles, as in that case early sedimentation prohibited the doughnut-hole formation. Furthermore, the effect of surface concentration and the way the drying process affects the internal structure of the drying droplet has been studied by means of microbeam small-angel x-ray scattering (SAXS) on a hanging droplet of a colloidal suspension containing silica nanoparticles of ~10-nm diameter. These experiments showed that isotropic assembly is still possible for Péclet numbers significantly higher than one and an accumulation of silica at the droplet surface was only observed for high initial concentrations [104]. This observation is to be expected, as shell accumulation may only occur if the colloid movement by diffusion is slower than the rate by which the droplet surface is receding due to evaporation. 

**Figure 9:**
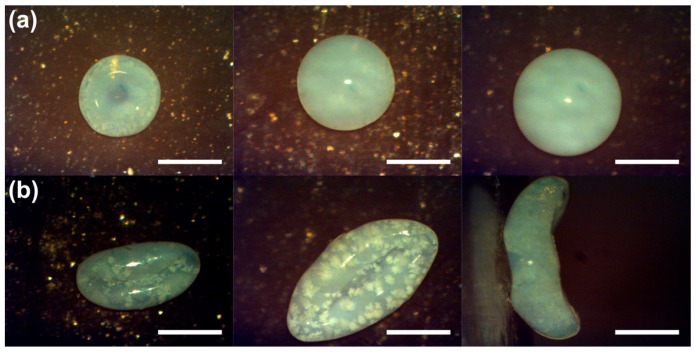
Examples for anisometric supraparticles obtained from drying fumed silica (FS) suspensions (from left to right 1.75%, 3.50%, 5.25% *w*/*v*) for an initial ionic strength of (**a**) 0.001 mM and (**b**) 25 mM using NaCl; the last image on the right side shows a side-view perspective. The scale bars are 500 µm. Adapted with permissions from [93,96] (pp. 587, 598). Copyright 2014 Wiley.

The symmetry of the supraparticles can substantially reduced be further towards anisometric, ellipsoidal shapes when fumed silica (FS) is used in aqueous colloidal suspension droplets instead of compact spherical silica nanoparticles. In contrast to the latter, FS has a very open and fluffy fractal structure of polydisperse particles with hydrodynamic radii of 100–200 nm and correspondingly large specific surface areas that typically are in the range of 50–400 m^2^/g [105]. However, the formation of anisometric supraparticles is only observed once a certain concentration of salt is present and the extent of anisometry can be controlled by adjusting the ionic strength [93,96]. Typical examples for different FS particle concentrations are illustrated for the case of no deformation in Figure 9a at 0.001 mM and for the maximum anisometry in Figure 9b obtained at 25 mM ionic strength of the suspensions at the start of the drying process.

Similar to previous findings for doughnut-shaped supraparticles, particle accumulation at the water-air interface occurs. This leads to a modification of the surface rigidity, which depends on the ionic strength, resulting in a higher rigidity for higher ionic strength. This could also be verified using time-resolved confocal laser scanning fluorescence microscopy. By labeling the FS and the water phase with different fluorescent dyes, the density profiles of the FS perpendicular to the droplet surface, i.e., in the radial direction, could be deduced throughout the drying process. Thereby, it was shown that the FS particles accumulate at the droplet surface and that the deformation of the droplet takes place once a certain effective thickness and density of this shell is reached [60]. In a somewhat related study, the evaporation process of an aqueous lysozyme solution on a superhydrophobic PMMA surface has been followed by microbeam SAXS. The evaporation process leads to hollow spherical residues, and the SAXS experiments show the increasing concentration at the droplet surface which leads to the precipitation of crystalline lysozyme nanoparticles towards the end of the drying process [106].

Coming back to the formation of the anisometric supraparticles, the main difference between the spherical silica microspheres used before and the amorphous ones of FS is the capability of intercalating and interconnecting during their aggregation, wherein this capability does not apply for spheres. In turn, FS can interact in a much more pronounced and cohesive way than silica spheres, where this attractive interaction will become dominant for sufficiently strong electrostatic screening. With increasing ionic strength and thereby lower Debye-screening length, the electrostatic repulsion between the FS particles becomes reduced. As an effect thereof, the shell rigidity arising from the intercalated FS particles at an ionic strength of above 0.5 mM increases such that the droplets can no longer shrink, while at the same time retaining their spherical shape. Accordingly, they become anisometrically deformed, just in the same fashion as a (spherical) football changes its shape when becoming deflated. The deformation leads to a droplet elongation, whose direction varies statistically, as it depends on the weakest spots of the as-formed shell. The resulting supraparticle anisometry, i.e., the ratio of principal axes lengths, could be precisely tuned within the range of 0.5–25 mM of ionic strength, and the observed anisometry is directly proportional to the logarithm of the ionic strength and reaches values of up to 1.6 [93,96]. A closer look into the formation mechanism revealed that the deformation occurs at a constant surface excess concentration of FS [95] and for an effective shell-thickness of about 22 µm, which corresponds to an interfacial FS volume fraction of 0.17 [60]. It might be noted that shell formation during evaporation can be related to buckling of the drying droplets. For instance, for µm-sized silica supraparticles obtained by spray-drying, the formation of doughnuts and dimpled/grainy spheres of buckyball appearance has been attributed to such surface instabilities. The buckling phenomenon could be arrested by the addition of PEO, which then allows for shape control [107].

**Figure 10:**
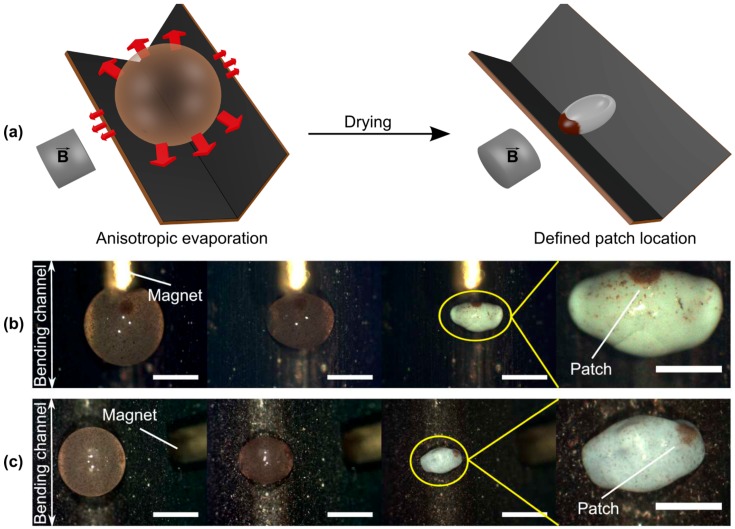
Controlled supraparticle orientation after drying: (**a**) anisotropic evaporation due to the surface bending. This allows for the creation of patchy anisometric particles with defined location of magnetic components (Fe_3_O_4_) by appropriately placing external magnets: (**b**) along the transversal or (**c**) longitudinal diameter of the ellipsoid. In (**b**) and (**c**), the images on the right side present zoomed-in excerpts; scale bars = 0.5 mm; other scale bars are 1 mm. Composed with permission from [108] (p. 5). Copyright 2016 Wiley.

The major drawback of producing anisometric supraparticles on flat surfaces is their statistical distribution of orientation after drying with respect to the surface placement. This renders it impossible to position a patch in a controlled fashion, as for instance is possible by using additional magneto-responsive ingredients within the precursor suspension droplets [92]. In a recent approach the process of anisometric supraparticle formation using FS [93,95,96] was modified by manipulation of the evaporation conditions using distinct surface geometries of the solid substrate [108]. More precisely, similar superhydrophobic substrates as utilized in the original experiments were bent at different angles into a V-shape. Monitoring the evolution of the droplet shape, a directed anisotropy perpendicular to the surface’s bending axis was observed. This effect of orientation is caused by the evaporation rate being higher at the free sides of the droplet compared to the blocked ones. This anisotropic evaporation, indicated by the red arrows in Figure 10a, leads to more accumulation of FS particles, which at ionic strengths above the threshold of 0.5 mM promotes the controlled droplet elongation perpendicular to the bending axis. It might be noted that this directional orientation then is reproducible with high precision, i.e., within a few degrees with respect to the bending axis of the surface. With this well-predictable orientation of the supraparticles it is possible to position patches of colloidal assemblies at will at any point(s) of the as-produced supraparticle (e.g., for magnetic colloids by an external magnetic field, but also other external influences could be imagined). Thus, this achievement constitutes a substantial advance for accessing increasingly more complex and functional supraparticles.

Accordingly, besides a general improvement of the extent and reproducibility of the anisometry values, by taking advantage of the predictable orientation of the final anisometric supraparticles, it is possible to render them patchy in a sophisticated and defined manner. For instance, supraparticles with distinct patch locations can be obtained by employing additional magnetic colloidal components (Fe_3_O_4_) and positioning an external magnet parallel (Figure 10b) or perpendicular (Figure 10c) to the bent channel. Such anisometric patchy supraparticles can be of potential use for designing self-propelling systems or applications requiring orientation functionality.

As already described in Section 2, Picknett and Bexon discussed the sessile droplet evaporation on solid surfaces using Maxell’s approach [41,42]. This also explained the anisotropic evaporation of the droplets on the V-shaped surfaces by introduction of an apparent CA, created by the upwardly-directed surface legs. Comparing the values for both, the surface and apparent CA obtained by fitting the CCA-model to the experimentally-obtained evaporation rate ratios (perpendicular vs. parallel to the surface bending axis), yielded a good correlation with the measured CAs. This revealed that the additional blockage by the upwardly-bent surface significantly reduces local evaporative vapor flux. Hence, anisotropic particle accumulation is promoted, being more pronounced on the free droplet faces, which in turn causes the controlled supraparticle elongation. 

**Figure 11:**
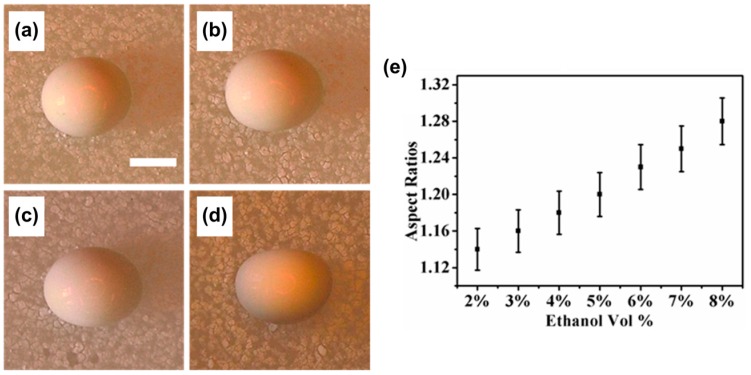
Optical microscopy of anisometric photonic crystals (PC) obtained by using mixtures of water and ethanol with varying compositions: (**a**) 2; (**b**) 4; (**c**) 6 and (**d**) 8 vol % of EtOH; scale bars are 800 µm. The systematic dependency of aspect ratios, i.e., anisometry values, is given in (**e**). Adapted with permission from [109] (p. 22647). Copyright 2015 American Chemical Society.

It might be worth noting that the formation of the anisometric FS supraparticles occurs in a non-pinned state, i.e., CCA-mode. However, Zhou et al. discovered a method that applies controlled pinning of the droplet contact-line using an ethanol-water mixture containing PS nanoparticles. This led to anisometric receding of the TPCL, producing anisometric photonic crystals [109]. By successive increase of the ethanol content, it was shown possible to create anisometric supraparticles within an aspect ratio from 1.14 (2 vol % EtOH) to 1.28 (8 vol % EtOH), as shown in Figure 11 [109]. This is an alternative approach to the FS supraparticle production, where droplet elongation occurs due to shell deformation [93,96,108]. Moreover, in pure aqueous systems, the structure of the resulting supraparticles could be tuned from microbeads to dimpled or microwelled shapes by varying the colloid concentration. 

Concluding, one may state that there exist different ways of symmetry breaking that lead to many quite different shapes, which are of interest for various potential applications.

### 5.2. Substructuring of Supraparticles

When using the droplet templating method on superhydrophobic surfaces, though the overall geometry may be fixed, still internal structuring or porosity may be of potential interest. One well-known structural type within this context is that of Janus or, more generally, patchy, hierarchical- or surface-anisometric particles, respectively [110,111,112,113]. Accordingly, we want to address supraparticles having different types of colloidal constituents that are inhomogeneously distributed throughout the assembled supraparticles.

**Figure 12:**
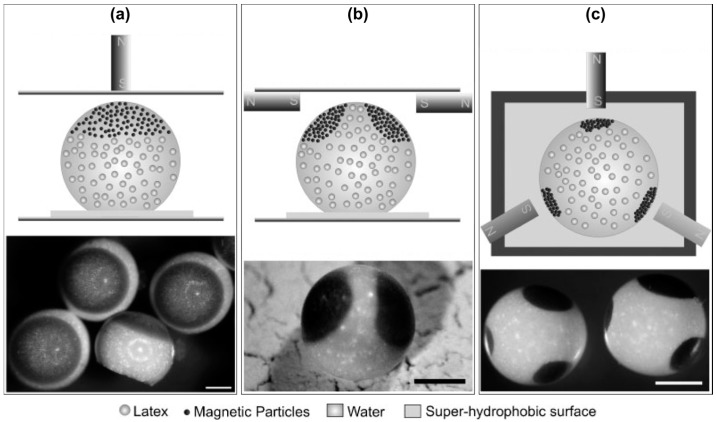
Assembly of (multi-)patched supraparticles in drying sessile droplets on a superhydrophobic surface: (**a**) single- (**b**) bi- and (**c**) tri-patched; scale bars are 500 µm. Altered with permission from [92] (p. 193). Copyright 2010 Wiley.

One straightforward way to achieve this kind of anisotropy in drying droplets is the utilization of magnetic components and the application of an external magnetic field [92,94,108]. The principle is shown in Figure 12, wherein besides the basic concept of forming single-patched supraparticles (Figure 12a), altering the magnetic field setup also allows for more complex patchy assemblies, such as presented by bi- and tri-patched supraparticles (Figure 12a,b).

Similarly, this patch formation can also be combined with anisometrically shaped supraparticles, as already described before and presented above in Figure 10b,c [108]. The combination of anisometric shape and controlled placing of patches on such particles in turn leads to much more complex supraparticles.

**Figure 13:**
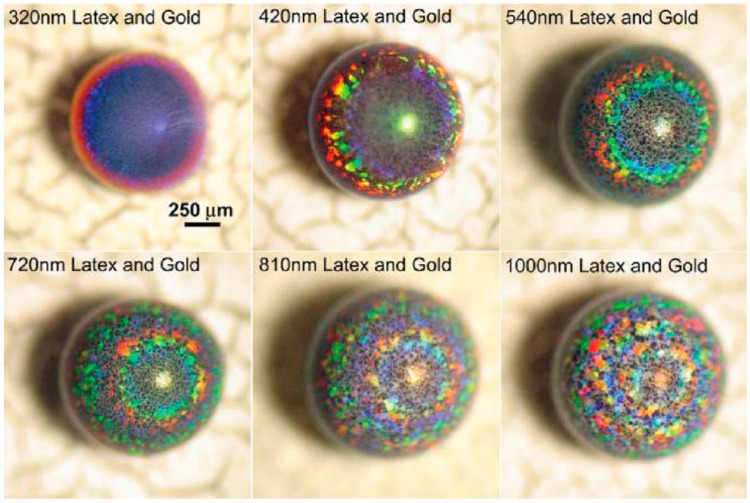
Optical microscopy images of patchy supraparticles assembled by EISA using variably-sized polystyrene (PS) latex nano-/micro-particles in suspension droplets generating highly light diffracting “opal balls”. The gold nanoparticles are 22 nm in size. Reprinted with permission from [97] (p. 4266). Copyright 2008 Wiley.

It is not necessarily required to use magnetic colloids with subsequent magnetic field guiding to generate hierarchically anisotropic, i.e., patchy particles. Rastogi et al. showed that by combining latex particles with diameters larger than ~300 nm (which collect on the droplet surface) with small gold nanoparticles (~22 nm), supraparticles, which exhibit remarkable optical features, as shown in Figure 13, can be obtained [97].

The surface collection of the suspended particles was found to arise from a combination of Marangoni flow, already discussed in Section 2, and the comparably small size of the gold nanoparticles. Due to the droplet evaporation taking place mostly at the top (surface blockage at the bottom), the temperature gradient promotes Marangoni flux transporting particles to the top. Using very low concentrations of PS microspheres, Chang and Velev investigated this effect inside an aqueous droplet floating in a fluorinated oil [33]. In these droplets, even though the particles were allowed to sediment, they again collected at the droplet surface after restarting the evaporation (by removing the saturated atmosphere). Thereby, the consequence of the Marangoni effect/flow was visualized. Accordingly, if the concentrations are properly set and because the gold nanoparticles are small enough to travel in-between the inter-particle separations of the bigger microspheres, the resulting supraparticles are rendered patchy with the gold particles collected at the top, as shown in Figure 13 [97]. Of course, by that method, it is also possible to cover a well-defined surface area of the final supraparticles. This effect is independent of the supraparticle shape, as exemplified by the “glazed doughnuts”, already presented in Figure 8 [92]. In summary, here, one faces a competition between sedimentation and induced Marangoni flow to which the dispersed colloids respond in a fashion depending on their size and density. This then leads to their segregation within the supraparticles and the observed internally inhomogeneous structuring, which allows for the creation of interesting and versatile supraparticles.

## 6. Applications of Supraparticles

As the preparation method leading to the final assembled supraparticles is not necessarily linked to their potential field of application, we will now extend our original focus and refer to supraparticles in a more general context. Nevertheless, for a start, we may first review some applications that emerged for supraparticles of the kinds described so far.

One example of potential applications is the field of photonics. Photonic applications require the precise control of optical properties of the materials, which can be achieved by proper nano-structuring. Defined spacing of monodisperse particles in highly ordered lattices allows for the diffraction of discrete wavelengths of the incident light leading to distinct coloring of the materials. Examples of this effect of defined coloring due to microstructuring can also be found in nature. Namely, the wings of *Morpho peleides* (butterfly) show an intense blue color without the presence of any dye [114]. Another example from nature is represented by the blue-purple fruits of an African tropical plant, named *Pollia condensata* [115]. Practically, assembling monodisperse particles into supraparticles with controlled inter-particle spacing allows for the fabrication of materials having tuned optical properties.

**Figure 14:**
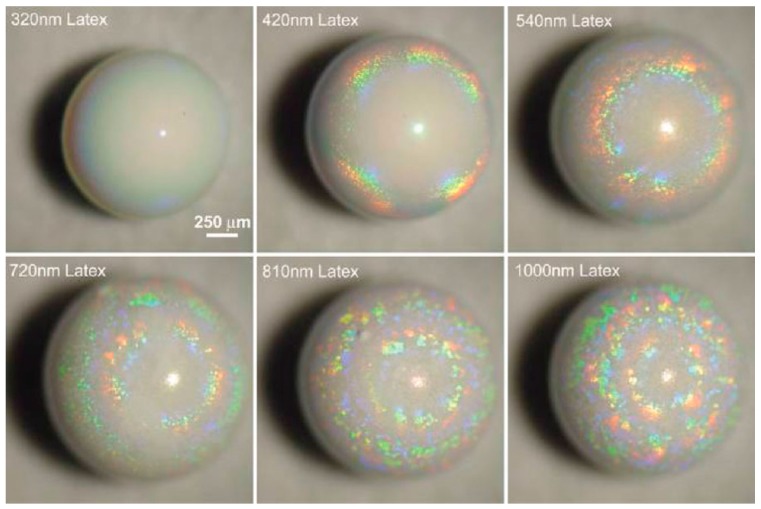
Optical microscopy images of patchy supraparticles assembled by EISA using differently-sized polystyrene (PS) latex microparticles in suspension droplets, thereby generating these highly light-diffracting “opal balls”. Reprinted with permission from [97] (p. 4266). Copyright 2008 Wiley.

As a first example, Rastogi et al. used PS microsphere suspensions in drying droplets on a superhydrophobic surface and varied the diameter of the PS particles between 320 and 1000 nm [97]. After drying, the resulting spherical “opal balls” show colored rings originating from diffracted light from the curved supraparticle surface (Figure 14). This diffraction is described by Bragg’s law, but rather than being caused by the colloidal crystal lattice within the bulk, it is more likely the result of the parallel rows at the surface. Furthermore, it was shown (see above in Figure 13) that additional gold nanoparticles of about 22 nm in size were not affecting the reflected colors, except for amplifying their intensity. Their color patterns can be precisely controlled by inter-particle spacing, which in turn is determined by the size of the microparticles. Of course, here, one may imagine even much more complex optical systems that could be achieved by appropriately designing hierarchical structures and using different colloidal components.

**Figure 15:**
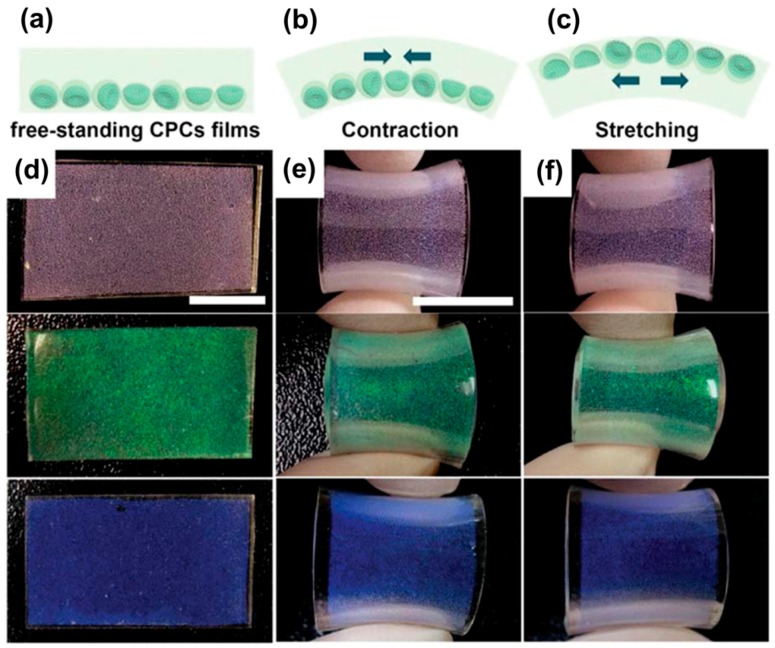
Colloidal photonic crystals (supraparticles) embedded in an elastomeric matrix in: (**a**) free; (**b**) contracted; (**c**) stretched state. Photographs of the resulting films are shown in (**d**–**f**) with supraparticles made of differently-sized PS particles. The reflected color is independent of the observing angle and strain on the films. The scale bars are 1 cm. Reprinted with permission from [116] (p. 1587). Copyright 2015 Royal Society of Chemistry.

Supraparticles showing optical effects can be employed to produce colored films or layers. Such films containing buckled or spherical supraparticles show angle and strain independent reflections due to the colloidal matrices provided by the supraparticles. This leads to observable structural coloring [116]. Such films can be obtained from emulsion droplets using osmotic annealing and defined salt-concentration to produce buckled or spherical supraparticles and entrapping them in a silicon matrix. The resulting films are presented in Figure 15d–f.

Here, the angle and strain independence is simulated by free-standing (Figure 15a), contracted (Figure 15b) and stretched (Figure 15c) films. These films show similar structural coloring arising from the supraparticles embedded into the silicon matrix, where buckled supraparticles showed even better color quality than spherical ones. 

**Figure 16:**
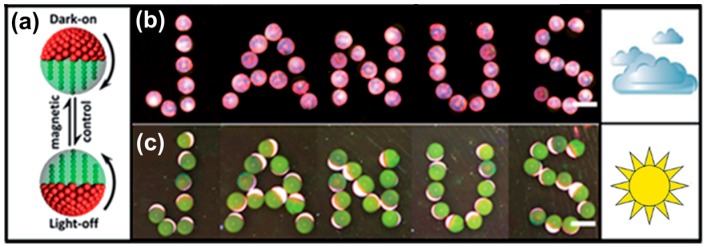
Magnetic Janus supraparticles (**a**) switched using the different hemispheres at different light intensities: upwards directed (**b**) PS hemisphere at low light intensity or (**c**) Fe_3_O_4_-TMPTA hemisphere under strong light intensity; scale bars are 500 µm. Adapted with permission from [117] (p. 9435). Copyright 2014 Royal Society of Chemistry.

Switchable photonic materials may be of potential use for displays, indicators or similar devices. As an example for such a device, Janus supraparticles having hemispheres of different reflectance with one of them being magnetic were produced using microfluidics [117]. These supraparticles then can be oriented with an external magnetic field (Figure 16a) showing different reflected intensity for different light conditions (Figure 16b,c), i.e., forming a magnetically-switchable display.

Similarly, also thermo-sensitive Janus supraparticles can be used in order to fabricate color changing displays [118].

**Figure 17:**
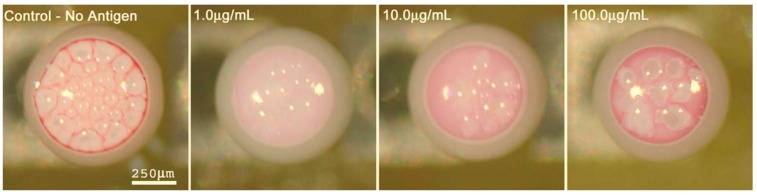
Resulting droplets for a 30-min incubation time and containing anti-rabbit IgG antibody functionalized gold nanoparticles at different concentrations of antigen (rabbit IgG); left to right: 0, 1.0, 10.0, 100.0 µg/mL. Reprinted with permission from [119] (p. 6). Copyright 2007 AIP Publishing.

Another interesting application of supraparticles is the development of bio-assays for antigen detection, which take advantage of the optical appearance of the supraparticles after drying depending on the exposure to the antigen and its concentration. Rastogi and Velev prepared an on-chip setup by drying droplets floating in fluorinated oil and containing PS microspheres and gold nanoparticles that were additionally functionalized with anti-rabbit IgG antibodies. Via the optical appearance after incubation, this system was able to serve as a sophisticated micro-bioassay for antigen detection [119]. This is a consequence of the antibody-antigen interaction, which is highly effective in terms of strength and specificity, thereby in turn controlling the particle interaction inside the dry droplets and consequently the resulting optical appearance. In other words, due to the antibody-functionalized gold and PS-latex particles, the patch formation is highly influenced by the presence of the corresponding antigen due to agglutination of the gold nanoparticles within the suspension. This effect also allowed for quantitative interpretation by optical comparison, shown in Figure 17 [119]. 

Not only for diagnostic, but also for therapeutic purposes, supraparticles can serve as drug delivery systems [120,121,122]. This can be achieved by incorporating drugs meant to be released over an extended period of time. It has been shown by Rastogi et al. that supraparticles are permeable for solutes and able to continuously release contained solutes in a very controllable way [87]. Thus, taking advantage of analogous mesoporous structures made of silica and gelatin hybrids, it was shown that brain-derived neurotrophic factor (BDNF), a protein growth factor, and dexamethasone (DEX), a steroidal anti-inflammatory drug, could be easily loaded into the supraparticles and continuously be released over several days [120]. Using this concept, BDNF-loaded supraparticles were implanted into the inner ears of guinea pigs previously deafened by frusemide and kanamycin medication (damaging ion gradients between the auditory neuron network). The release of BDNF thereby showed significant rescuing of primary auditory neurons, potentially preserving residual hearing [121]. In another experiment, Park et al. immobilized a cysteine protease to porous calcium phosphate supraparticles [122]. This protease-supraparticle hybrid, in vitro allowed for systematic inactivation of the cytokine tumor necrosis factor-alpha (TNF-α), which is responsible for inflammatory effects potentially causing autoimmune diseases.

**Figure 18:**
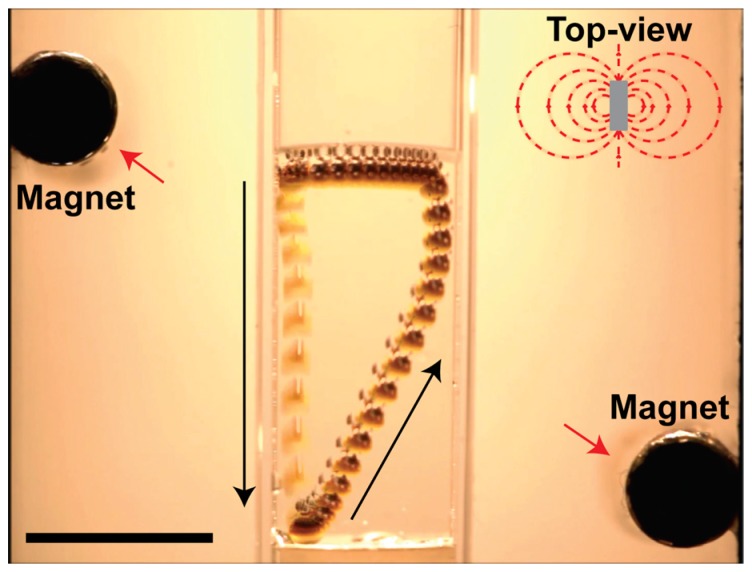
Oscillating elevator supraparticle in a wt % aqueous H_2_O_2_ solution. Starting at the top left, the elevator releases the oxygen bubble and falls down to the left bottom. After producing an oxygen bubble, it gets attracted by the bottom right magnet, following this attraction to the right during the way up. Having reached the surface meniscus, the left magnet pulls the elevator supraparticle back to its starting position, restarting the movement cycle; the scale bar is 1 cm. Reprinted with permission from [94] (p. 6). Copyright 2016 Wiley.

A quite different functionality of supraparticles is their potential ability to move. Self-propelling particles convert chemical energy into active, autonomous motion and have been developed and built in many different ways [123,124]. Able to perform complex tasks, such artificial vehicles have promising potential for applications in environmental treatment, like water remediation [125,126] or can serve as smart drug-delivery systems [127]. Yet, an interesting approach extending the field towards the millimeter scale has been taken using supraparticles prepared by EISA on a superhydrophobic surface [94]. These supraparticles were rendered patchy using Fe_3_O_4_ core nanoparticles decorated with Pt and a magnetic field during the synthesis. After additional surface hydrophobization and when placed in a H_2_O_2_ solution, these supraparticles generated adhering oxygen bubbles (for sufficient adhesion, the hydrophobization is essential), which increased the buoyancy. Once the buoyancy is high enough (typically one larger oxygen bubble is attached to the supraparticle), the whole supraparticle is lifted to the liquid’s surface. There, it loses its oxygen bubble and drops down to the bottom of the liquid again, where the formation of a new bubble starts. The whole process then is repeated, and the supraparticle performs an oscillating, regular vertical motion, thus resembling “elevators”, and this motion can continue for days. In addition, via the contained magnetic nanoparticles, the trajectory of the supraparticle can be steered by the application of an external magnetic field, which is illustrated in Figure 18.

Functionalizing the elevator supraparticle surface with the enzyme α-amylase, the catalytic decomposition of starch could be performed as a model reaction, showing potential applications of these elevator supraparticles in chemical catalysis [94]. Accordingly, this example proves the concept of self-propelled particles, whose movement can be steered by an external magnetic field and which are able to perform a chemical task (reaction) on their trajectory. Of course, the use of H_2_O_2_ limits the potential for applications substantially, but it might be noted that it is also possible to use more benign, non-toxic fuels, such as alcohols, for particle propulsion [128]. 

Finally, supraparticles are also interesting for the general field of catalysis, as they constitute rather versatile and small reaction containers. As an example in the field of catalysis, zinc sulfide supraparticles were developed for chemical dechlorination of 2,2′,4,4′,5,5′-hexachlorobiphenyl, an organic pollutant occurring in soils or groundwater [129]. Using UV irradiation, these supraparticles reductively degraded the model pollutant up to about 70% after 12 h in a solution of isooctane. Even more complicated, Xu et al. showed that Pt nanoparticles bound to a porous organo-polymer shell synthesized via soap-free emulsion polymerization onto Fe_3_O_4_ supraparticles can perform catalytic enantioselective hydrogenation of ethyl pyruvate [130].

Of course, this short section is far from complete with respect to the applications that have been explored for supraparticles, simply due to the fact that this is an ample field of research that allows for a vast number of options via appropriate structural and functional design by using various kinds of colloidal components that can be readily applied. Thus, many interesting developments have already been achieved and many more are to be expected in the near future to emerge from this field. 

## 7. Conclusions

Supraparticles can be rich in terms of their size, shape and internal structure, and their properties can be varied largely via the choice of their colloidal constituents. In our review, we focused deliberately on supraparticles in the size range of hundreds of µm or even mm. Of course, colloidal assembly is not limited to this size range, and a much work has also been done on the assembly of nanoparticles, including their structured assemblies at surfaces [6,131,132,133,134]. However, in this review, we explicitly refrained from covering such investigations.

For the fabrication of supraparticles, a large number of methodologies has been developed, where in particular evaporation-induced self-assembly (EISA) on superhydrophobic surfaces has distinct advantages, which therefore was also the focus within this review. First, it is rather simple to collect the pure supraparticles subsequent to their synthesis since no other solvent has to be removed. Secondly, it has been shown that via EISA, one can achieve anisometric supraparticles whose orientation can be controlled by appropriately structuring the superhydrophobic substrate. This consequently allows preparing anisometric patchy supraparticles where the location of the patches can be precisely controlled (for instance for patches containing magnetic nanoparticles by an external magnet). It might be noted that this approach is not limited to the use of aqueous colloidal dispersions. Instead, the utilization of superamphiphobic surfaces also allows for its extension to a large range of organic solvents.

By combining the shape control of the supraparticles with a detailed control over the internal, e.g., hierarchical, structure by careful choice of the colloidal components, increasingly more complex systems with tailored functionality are available, which are interesting for a multitude of applications. These functionalities include optical properties that can be interesting for photonic applications and electric or catalytic properties that can be achieved by incorporating appropriately active (nano-)particles. Another functional feature is self-propulsion, which for instance can be achieved by incorporating nanoparticles and/or enzymes able to induce chemical reactions that lead to a momentum on the supraparticle. The movement then can be of a vertical and/or horizontal direction and also magnetically steered. One may also combine such a movement with other functional properties additionally incorporated in these supraparticles, such as catalytic activity for performing chemical reactions, thus providing “chemical microrobots”. These “multi-tasking” supraparticles can be expected to become substantially further developed, and one may envision almost an unlimited potential via the combined functionalities that can be imparted. 

In summary, there are rather simple ways to produce supraparticles, and by appropriate design, it is possible to integrate different functionalities into them, which can be independently combined and addressed. Accordingly, they represent smart systems, able to exhibit many interesting properties and being useful for several applications, like optical, electronic, chemical, mechanical ones, etc. However, the state of the art in this area certainly is still in its infancy, and one may expect many interesting developments leading to increased complexity in the structure and function of future materials.

## Abbreviations

BNDF	brain-derived neurotrophic factor
CA	contact angle
CCA	constant contact angle
CCR	constant contact radius
cmc	critical micelle concentration
CVD	chemical vapor deposition
DEX	dexamethasone
ECD	electrochemical deposition
EISA	evaporation-induced self-assembly
FS	fumed silica
PEO	polyethylene oxide
PMMA	poly methyl methacrylate
PS	polystyrene
SEM	scanning electron microscopy
TMPTA	trimethylolpropane triacrylate
TNF-α	tumor necrosis factor-alpha
TPCL	three-phase contact line
UV	ultra-violet
WCA	water contact angle
